# Validation of a Novel RP-HPLC Technique for Simultaneous Estimation of Lignocaine Hydrochloride and Tibezonium Iodide: Greenness Estimation Using AGREE Penalties

**DOI:** 10.3390/molecules28083418

**Published:** 2023-04-13

**Authors:** Sana Hanif, Muhammad Ali Syed, Ahmad Junaid Rashid, Tareq Nafea Alharby, Mohammad M. Algahtani, Muteb Alanazi, Jowaher Alanazi, Rai Muhammad Sarfraz

**Affiliations:** 1 College of Pharmacy, University of Sargodha, Sargodha 40162, Pakistan; pharmacist.sas22@gmail.com; 2 Faculty of Pharmacy, The University of Lahore, Lahore 54590, Pakistan; 3 Department of Pharmaceutical Sciences, Faculty of Chemistry and Life Sciences, Government College University Lahore, Lahore 54000, Pakistan; 4 Quality Control Department, Pacific Pharmaceuticals Limited, Lahore 54000, Pakistan; 5 Department of Clinical Pharmacy, College of Pharmacy, University of Hail, Hail 81442, Saudi Arabia; 6 Department of Pharmacology and Toxicology, College of Pharmacy, King Saud University, Riyadh 11451, Saudi Arabia; 7 Department of Pharmacology and Toxicology, College of Pharmacy, University of Hail, Hail 81442, Saudi Arabia

**Keywords:** tibezonium iodide, lignocaine, method development, ICH guidelines, AGREE, greenness

## Abstract

Herein, we reported an HPLC method for the simultaneous determination of tibezonium iodide (TBN) and lignocaine hydrochloride (LGN). The method was developed according to the International Conference for Harmonization guidelines (ICH) Q2R1 using Agilent^®^ 1260 with a mobile phase consisting of acetonitrile and phosphate buffer (pH 4.5) in a volumetric ratio of 70:30 and flowing through a C_8_ Agilent^®^ column at 1 mL/min. The results revealed that TBN and LGN peaks were isolated at 4.20 and 2.33 min, respectively, with a resolution of 2.59. The accuracy of TBN and LGN was calculated to be 100.01 ± 1.72% and 99.05 ± 0.65% at 100% concentration, respectively. Similarly, the respective precision was 100.03 ± 1.61% and 99.05 ± 0.48%. The repeatability for TBN and LGN was found to be 99.05 ± 0.48% and 99.19 ± 1.72%, respectively, indicating that the method was precise. The respective regression co-efficient (r^2^) for TBN and LGN was found to be 0.9995 and 0.9992. Moreover, the LOD and LOQ values for TBN were 0.012 and 0.037 µg/mL, respectively, while for LGN, they were 0.115 and 0.384 µg/mL, respectively. The calculated greenness of the method for ecological safety was found to be 0.83, depicting a green contour on the AGREE scale. No interfering peaks were found when the analyte was estimated in dosage form and in volunteers’ saliva, depicting the specificity of the method. Conclusively, a robust, fast, accurate, precise and specific method was successfully validated to estimate TBN and LGN.

## 1. Introduction

According to the World Health Organization (WHO), maintaining buccal health is an integral core component when defining the overall quality of health in order to minimize the risk of oral pathologies [1]. Oral cavity ailments may contribute pathological manifestations such as unhealed mouth ulcers, complicated or uncomplicated microbial infections, sore throat, dental caries and other similar clinical situations [2,3,4]. For infection-related situations, the use of antimicrobials and antiseptics are important to maintain sterility in the oral cavity. These strategies typically include treatment with antibiotics or combined either with NSAIDs, anesthetics or antiseptics to alleviate infection and the associated clinical symptoms [5]. However, the topical application of such drug protocols may reduce the dose of the drug and drug-related adverse reaction [6,7]. A number of antiseptics are commercialized as part of mouthwashes, lozenges, creams, gels or such other dosage forms to control microbial flora in such situations. 

**Figure 1:**
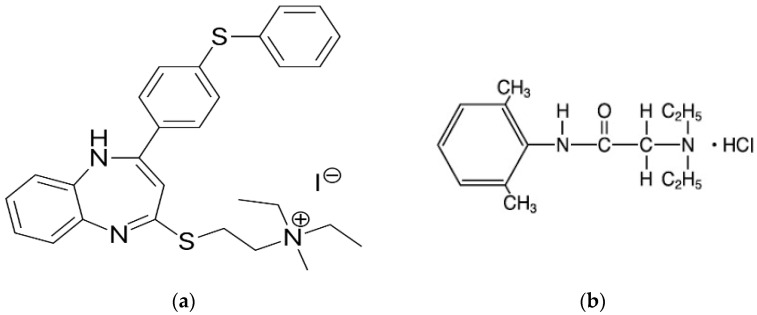
Chemical structures of (**a**) tibezonium iodide and (**b**) lignocaine hydrochloride.

Tibezonium iodide is one such agent that is used ‘over the counter’ as an antiseptic product. It is an antiseptic substance consisting of a lipophilic quaternary ammonium ion with iodide being the anion part, is a yellowish powder, and possesses antiseptic effects locally. It is marketed under the trade name of Maxius^®^/Tyzorin^®^ (mouthwash and chewable tablets) in different regions worldwide. The drug TBN is used to improve the clinical symptoms of mouth infections, bronchitis, gingival inflammation, dental plaque or symptoms similar to the common cold or influenza [8]. A dose of 5 mg is recommended to be administered every 2–3 h to maintain the antiseptic environment in the buccal mucosa and it is considered non-mutagenic at the mentioned doses [9,10]. The sustained delivery of the drug was reported in the literature for better therapeutic efficacy [8,10,11]. Being a larger molecule (Figure 1), TBN is insoluble in distilled water, mildly soluble in ethanol and soluble in methanol, acetonitrile and 0.25% *w/v* SLS solution [12]. Optionally, adding a local anesthetic to an antiseptic substance may cause synergistic effects with TBN by reducing the pain sensitivity towards oral soreness and ulcers [13].

Lignocaine hydrochloride (LGN) is a local anesthetic agent that is frequently employed in medicine and dentistry for its efficacy and safety compared to others in its class [14]. It appears as a white crystalline powder that is soluble in water and freely soluble in ethanol, methanol, acetonitrile and solutions of sodium lauryl sulphate [15]. Chemically, LGN is a mono isotopic small molecule (Figure 1) containing amide ester and possessing a molecular weight of 234.3 [16]. In topical products, it was safe and effective when delivered in a 5% gel with rare medication-associated risks. Lignocaine possesses analgesic efficacy as well [17].

Medicinally, both molecules (Figure 1) are used as therapeutic substances for treating sore-throat-related pathologies by local action. Both agents are commercialized as buccal lozenges [18]. However, their efficacy is enhanced when ‘over the counter’ substances are combined [19,20]. Previously, tibezonium iodide was combined with different local anesthetic substances in an attempt for better local drug concentration in the saliva [8,10,11,21]. Additionally, the frequency of administration was also reduced by maintaining a reasonable therapeutic drug concentration in saliva. This emphasizes that the combination of molecules as therapeutic substances are effective in improving the clinical symptoms of sore throat, for which it can be an effective strategy to combine lignocaine hydrochloride and tibezonium iodide for simultaneous delivery to combat the pathology [12]. Although, High-performance liquid chromatography (HPLC) methods for estimating LGN are reported in the literature, whether singly or in combination with other agents [22]. Previously, we reported an HPLC method for the estimation of TBN with another local anesthetic agent, benzocaine [12]. However, no reasonable method was found in the literature for the simultaneous estimation of TBN and LGN. Hence, we designed the current study to estimate TBN and LGN simultaneously in a mucoadhesive buccal drug delivery and human saliva. To accomplish this, the instrumental conditions were optimized. Then, the developed method was validated according to the criteria of the ICH guidelines [23]. After validation, the method was applied to estimate drugs in dissolution media as well as in the saliva of healthy volunteers. Eventually, the greenness of the validated method was found using AGREE^®^ and the penalty points were assessed [24].

## 2. Results and Discussion

Validation of the analytical method is an integral stage once a drug is developed or combined with another agent during the research and development stage of a product. It is an analytical technique to quantify the drug in dissolution media. The current method explored various parameters of validity for the simultaneous determination of TBN and LGN in combined dosage form and in human saliva, which was previously found in the literature to the knowledge of authors. The method was developed initially by testing under raw conditions, but it was optimized with conditions (Table 1) reported in the current study and validated as per the ICH guidelines.

### 2.1. Method Development and Optimization

Optimization was initially based on suitable conditions for peak separation on a trial basis. The absorption spectra of both drugs were evaluated in various solvents to obtain the maximum absorbance points across wavelength of 270, 242 and 210 nm of which 242 nm was found important to analyze both molecules after peak spiking. At the same time, the instrumental conditions such as the temperature of the column and the flow rate were optimized along with minor modifications of pH in the mobile phase to obtain better resolution and peak timings. 

With alcohols, it was experienced that minor interfering peaks were present at the retention time of LGN. Additionally, the retention time was augmented, for which acetonitrile was considered. Economically, the price of the solvent was also another factor while optimizing the mobile phase. When the organic ratio in the current mobile phase was reduced to enhance the greenness objective, shouldering in the peak of LGN was observed. The aqueous phase on the other hand caused augmentation in the retention of TBN when its ratio was incremented. TBN, being poorly soluble in the aqueous phase, always eluted after the LGN peak. Using the current reported HPLC conditions, two sharp peaks for TBN and LGN were recorded in the chromatogram with retention times of 4.2 and 2.3 min, respectively.

### 2.2. System Suitability

The results (Table 1) for system suitability shows that the system coincided with the standard for method development and all the measurements were within the range.

#### 2.2.1. Retention Time

Both peaks were identified within 5 min after the sample was injected, where the retention time for LGN and TBN were 4.20 ± 0.28 and 2.33 ± 0.18, respectively (Figure 1). In another study, TBN was delivered with another anesthetic substance, benzocaine, for local action where the peak time for TBN was approximately 4.15 min, which is near the peak in current study. The composition of acetonitrile in a reported study was also equal to the volume used in the current study for every mL [12]. This depicts that the composition of acetonitrile is important in maintaining the retention of TBN because the column was also different in that previously reported study. With respect to benzocaine, the retention time is also near to the retention of LGN since both drugs are soluble in water [25].

#### 2.2.2. Resolution

The resolution between the peaks was found to be 2.591, which showed that there was a lapse of more than a minute until the TBN was separated from the column mixture. This value is reasonable since, as the resolution increases, the method will become expensive and laborious.

#### 2.2.3. Tailing Factor

The tailing factor of each drug was less than 2, i.e., 1.86 and 1.32 for TBN and LGN, respectively, which is considered acceptable with respect to the Food and Drug Administration–Center for Drug Evaluation and Research (CDER-FDA) guidelines [26]. The more the tailing value is evident, the more the peak will take time to be eluted from the column and elongation of the tail will be evident due to asymmetry [27].

#### 2.2.4. Theoretical Plate Count

The theoretical plate count was found to be more than 2000 for both molecules, which is considered acceptable according to CDER-FDA guidance [26].

### 2.3. Method Validation

#### 2.3.1. Linearity

For determining the linearity range, the concentration peak response versus dose was plotted, which generated a range of linearity along with the values of regression (r^2^) and linearity equation (Table 1). Different concentrations of TBN and LGN were prepared in the current study to find linearity in the respective range of 0.14–10.08 and 0.28–20.18 μg/mL. The coefficient of linear regression (r^2^) for TBN and LGN was found to be 0.9995 and 0.9992, respectively.

#### 2.3.2. Accuracy

The accuracy was measured by preparing concentrations of both drugs at 80, 100 and 120% and then finding the recoveries where the procedure was repeated thrice to obtain the RSD (%) for each concentration. The results revealed that the recovery of drugs were in an accurate range and the relative standard deviation was within 2%. This confirms that the quantitative estimation of the drugs was accurate for the proposed method in the ranges prepared in the study. The recovery values along with the RSD (%) are given in Table 2.

#### 2.3.3. Precision

The precision relates to the similarity of the invented method’s response to the same concentrations when the test is conducted on different days, by dissimilar analysts and using different machines. It helps to ensure that the instrumental conditions of HPLC are unaffected by machine, day or personnel variations. The precision in terms of repeatability, intermediate accuracy and reproducibility was determined. Analytical concentrations (80, 100 and 120%) were tested on different days for repeatability and intermediate precision. The same instrumental conditions were also run on different machines to assess the reproducibility. The results revealed that the values of intermediate precision, repeatability and reproducibility were not significant and the RSD values (%) were less than 2%, which is generally considered acceptable [12], indicating that the method was precise (Table 3).

The analytical method development was accurate in calculating the amount of the drug and the calculations repeated on different days by different analysts predicted the preciseness and linearity in the stated concentrations. These findings are in accordance with the previous study reported, in which a different anesthetic agent was used with tibezonium iodide. Similarly, the results of the different parameters measured at different concentrations of both drugs showed that the relative standard deviation value was less than 2%, which showed that these findings met the ICH guidelines [23].

#### 2.3.4. Limit of Detection (LOD) and Limit of Quantification (LOQ)

The LOD is the minimum quantity of substance identified by the system, while the LOQ is the minimum quantity of the substance that can be calculated quantitatively using the developed method [28]. Generally, the LOD should be more than three times greater than the noise peak from the baseline. In case of TBN, the LOD and LOQ values were found to be 0.012 and 0.037 µg/mL, respectively. The found values of TBN were comparable with the findings reported in the literature [12]. For LGN, the values were 0.115 and 0.384 µg/mL, respectively (Table 1).

#### 2.3.5. Robustness

The robustness of the developed system was tested by measuring the product peak area against minor changes in the flow rate, temperature and pH. Changes in the flow rate, pH and temperature were 0.85–1.15 mL/min, 4.0–5.0 and 30–40 °C, respectively (Table 4). The results revealed that all changes in the HPLC conditions did not significantly alter the peak area RSD values (%) and the tests were within the limits in compliance with the ICH guidelines, i.e., less than 2%, reflecting the stability of the method.

#### 2.3.6. Specificity

**Figure 2:**
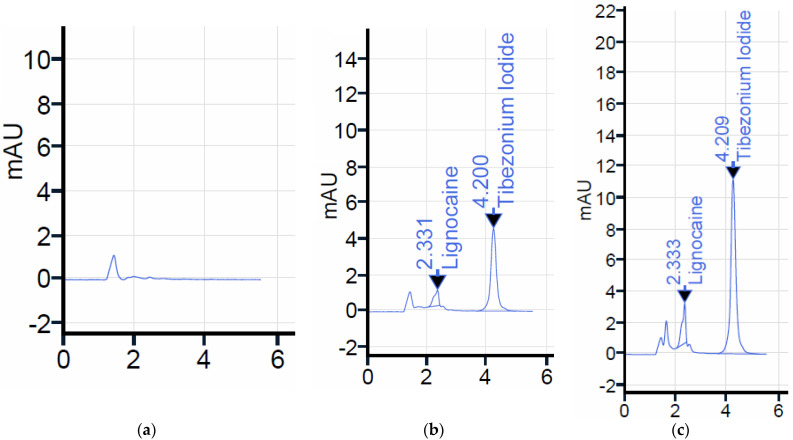
Chromatograms indicating (**a**) placebo dosage form depicting SLS peak, (**b**) drug peaks in the PBS, pH 6.8 and (**c**) human salivary sample analysis. The peaks of drugs in human salivary samples revealed no interacting peaks of the ingredients with that of the drugs. This confirms that the current method is suitable for the dual estimation of LGN and TBN.

No interfering peak was found in a blank sample (Figure 2c), pure drug samples (Figure 2b), pharmaceutical dosage form and human salivary drug concentrations (Figure 2c). It was observed that no additional peak or noise was observed in a chromatogram of the in vivo drug analysis. However, it is a limitation of the current study that the degradation studies should be carried out for both molecules under accelerated stability conditions. Since no reasonable data are present in the literature, it is an important area of development to conduct research, because TBN is a marketed drug and is available to millions of people worldwide.

### 2.4. In Vitro Dissolution Analysis

The chromatographic analysis of in vitro drug release revealed no interference or change in the timings of TBN and LGN. There was no additional peak of inactive pharmaceutical ingredients overserved, supporting that the approach can be used for simultaneous in vitro release measurement of TBN and LGN from the buccal mucoadhesive dosage form [10]. This might be due to the fact that the polymers used in the reported literature contained long chains and/or were water dispersible, for which the ingredients did not interfere with the soluble drugs in the dissolution fluid at pH 6.8.

### 2.5. Salivary Drug Concentrations in Volunteers

The salivary values were estimated according to the procedure reported in [29]. The sample was obtained by tilting the head of healthy human volunteers and collecting saliva using the sterile tip of a micropipette. The detailed protocol of the salivary volunteer sampling was reported in another study by the author [10]. The results depicted distinctive peaks of LIG and TBN (Figure 2c).

### 2.6. Greenness of HPLC Method

**Figure 3:**
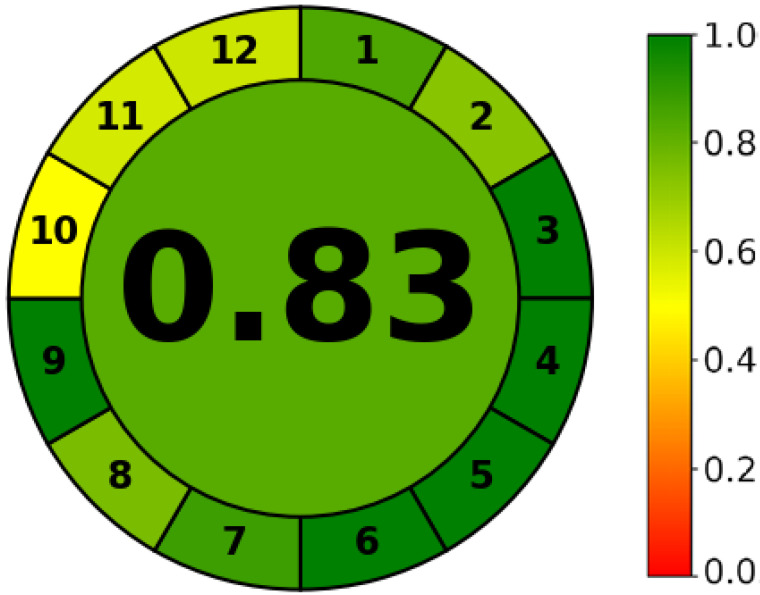
Greenness of the developed method using AGREE software.

The greenness ensures the environmental safety aspect of the validated method. The more the substances, chemicals, reagents, processes and steps involved in the developed method, the worse the environment will be affected. Therefore, it is of prime importance that the analysis should be an eco-friendly method [30]. There are different greenness evaluation software that display the detailed pros and cons of any method [30,31]. Nevertheless, AGREE displays penalties by contour shades near to or far from the ideal greenness by keeping in view the safety of workers as well. The calculated AGREE score of the developed method was found to be 0.83 due to the deduction of penalty points (0.17), since the organic concentration in the mobile phase is 70% *v*/*v* acetonitrile, which is expected to contribute slight toxicity to aquatic life and inflammability in nature (Figure 3). However, the retention of both drugs is approximately less than five minutes (Figure 2b) in a single run, which would theoretically be contributing less wastage of the mobile phase. The wastage of LGN was found more in literature, where the sample analysis time in a single run was time-consuming [32,33,34].

## 3. Materials and Methods 

### 3.1. Materials/Chemicals/Reagents 

Tibezonium iodide (Recordati^®^, Correggio, Italy), sodium lauryl sulphate (SLS), sodium dihydrogen phosphate, monobasic potassium phosphate, orthophosphoric acid and other chemicals/solvents used in the preparation of the mobile phases were generously donated by Pacific Pharmaceuticals Limited (Lahore, Pakistan). A parenteral grade Millipore^®^ filter was used before injecting the salivary and standard solution samples. Lignocaine hydrochloride was attained from Hoover Pharmaceuticals Private Limited (Lahore, Pakistan). Membrane-filtered reverse-osmosis water was used during the whole study unless otherwise mentioned.

### 3.2. Instrumental Conditions

The analytical method development was carried out on an Agilent^®^ (Machine 1260 Infinity^®^) HPLC system with VWD1A for UV detection. The machine was fitted with a C_8_ Agilent (150 × 4.6 mm, 5 μm) column and auto vial sampler G7129A injector preset at an injection volume of 10 μL. The machine also contained a quaternary Pump VL G7111A. To analyze the peak attributes, OpenLab^®^ software was used. After each auto-sampling, the injector needle was auto-washed with methanol. A similar instrument was used for interday testing operated by another analyst.

### 3.3. Mobile Phase Preparation

The mobile phase was prepared by mixing acetonitrile and 0.02 M monobasic potassium phosphate (pH 4.5, adjusted with orthophosphoric acid) in a ratio of 70:30 *v/v*, respectively. The mobile phase was degassed for 10 min by sonication and subsequently membrane-filtered using a vacuum pump before use.

### 3.4. Standard Solution Preparation 

The stock solution was prepared by carefully weighing 111.12 and 55.56 mg of LGN and TBN, respectively, in 500 mL of 0.25% *w/v* SLS, adjusted to pH 6.8 with potassium dihydrogen phosphate and sonicated for 10 min until completely dissolved. From this stock solution, different dilutions were prepared with the intention to construct a linearity range. The standard solution, representing 100% concentration of both drugs, was formed by dissolving 55.56 and 27.78 mg of LGN and TBN, respectively, in a similar SLS solution. Finally, 10 mL of this standard solution was diluted to 100 mL of 0.25% *w/v* SLS solution for analysis. 

### 3.5. System Suitability Parameters

For system suitability, the retention time of both peaks, resolution between the peaks, tailing factor of the peaks and theoretical plate count were calculated.

#### 3.5.1. Retention Time

The retention time of the peak was calculated from the arbitrary time of injection of the sample in the machine column and the time it was eluted out and detected with the ultraviolet detector. 

#### 3.5.2. Resolution

The resolution or separation between the peaks was measured using Equation (1):

(1) $$\mathrm{Rs}=\frac{\left(\mathrm{tR}2-\mathrm{tR}1\right)}{0.5\left(\mathrm{tW}1+\mathrm{tW}2\right)}$$

The resolution ‘Rs’ was directly dependent upon the difference between the retention time of drug 1 (tR1) and drug 2 (tR2). Rs was inversely related to the peak width of drug 1 (tW2) and drug 2 (tW2) [24].

#### 3.5.3. Tailing Factor

The tailing of the peak was measure using Equation (2) as follows.

(2) $$\mathrm{Tf}=\frac{a+b}{2a}$$

where in the above equation, the tailing factor ‘Tf’ was dependent upon ‘a’ and ‘b’. The ‘a’ represents the distance of the peak of the drug from the frontal half-side to the mid-point of the peak and is evaluated at 5% altitude. Similarly, ‘b’ refers to the length from the center of the peak to the trailing side of the peak (estimated at 5% peak height) [35].

#### 3.5.4. Theoretical Plate Count

The theoretical plate count according to the United States Pharmacopeial (USP) guidelines is stated using Equation (3):

(3) $$N=\left(\frac{\mathrm{tR}}{\sigma }\right)\cdot \left(\frac{\mathrm{tR}}{\sigma }\right)$$

where ‘N’ is the plate count, ‘tR’ is the retention time of the peak considering a normal distribution and ‘σ’ is the standard deviation of the peak [36]. 

### 3.6. Validation Parameters for the Proposed Method

After the instrumental conditions were evaluated for system suitability, it was further tested for validation parameters according to the ICH Q2(R1) guidelines. These included the linearity, accuracy, precision, limit of detection, limit of quantification, robustness and specificity [23]. To evaluate the magnitude of the deviation from the given parameters, all the parameters were evaluated thrice and the results were expressed as an average of the relative standard deviation. 

#### 3.6.1. Linearity

The calibration curve was constructed from the stock solution (explained in Section 3.3) in such a way that a series of dilutions was prepared to evaluate the linearity range of both drugs [37]. 

#### 3.6.2. Accuracy

The accuracy was evaluated by calculating the percentage recoveries of the standard solutions. It was deliberately performed in terms of percent recoveries of analytical molecules when the concentration was kept at 3 levels at 80, 100 and 120% *w/v* [35]. 

#### 3.6.3. Precision

The precision was measured on three concentrations as mentioned above under the accuracy procedure. Additionally, the analytical concentrations prepared were analyzed on day one by the analyst using an Agilent^®^ machine. When the repeatability was evaluated, different analysts determined the drug contents on a different machine on a different day and at least three samples were tested against each concentration [35]. 

#### 3.6.4. Limit of Detection (LOD) and Limit of Quantification (LOQ)

The LOD and the LOQ were identified by dividing the standard deviation (Sy) value obtained from the lowest concentrations of analyte prepared for determining the linearity range with the slope of the linearity line (S). Equations (4) and (5) were used for estimating LOD and LOQ parameters, respectively, for TBN and LGN [38].

(4) $$\mathrm{LOD}=\frac{\mathrm{Sy}}{S}$$

(5) $$\mathrm{LOQ}=\frac{\mathrm{Sy}}{S}\times 3.3$$

#### 3.6.5. Robustness

Similarly, the robustness was analyzed by considering minute changes in the pH, temperature of the column as well as mobile phase flowing through the column and the response was recorded [35]. The samples were tested thrice for estimating the RSD (%) values.

#### 3.6.6. Specificity

Ultimately, the specificity of the method was assessed to check the probability of intervening peaks at the retention times of standard solutions of drugs, dosage form and human salivary samples. For assessing volunteers’ saliva, favorable ethical opinion was attained from the Committee of the Institutional Review Board for assessing the concentration of salivary drugs in healthy human volunteers (REC/DPP/FOP/6A). Concisely, saliva was collected from healthy volunteers and diluted with the solution of the in vitro dissolution media, i.e., 0.25% SLS, according to the protocols described in Section 3.8 [29]. 

### 3.7. In Vitro Dissolution Studies

The in vitro dissolution studies of the buccal mucoadhesive dosage form were carried out by employing a USP type II paddle apparatus at a speed of 50 rpm at 37.5 ± 0.5 °C throughout the experiment [7]. The dissolution media comprised 900 mL of 0.25% *w*/*v* SLS solution, adjusted to pH 6.8. Samples of 5 mL were withdrawn at intervals of 0.5–6 h, which were then clarified with a Millipore^®^ filter membrane and directly run onto HPLC for analysis. Fresh dissolution media was added after each sampling to replenish the volume [6].

### 3.8. Salivary Drug Concentration

The in vivo drug concentrations were tested only on an optimized mucoadhesive buccal formulation for the estimation of concentrations of TBN and LGN in vivo [10]. To accomplish this, favorable ethical opinion was attained from the Institutional Review Board (IRB) from the University of Lahore prior to conducting the experiment (REC/DPP/FOP/6A) pertaining to the single-dose application of the dosage form and sampling of saliva of healthy volunteers. During the experiment, the ethical guidelines and protocols of the Declaration of Helsinki were followed.

The procedure and protocols to find the salivary drug concentration in volunteers was followed as reported in the literature [29]. Briefly, with the sterile tip of a micropipette, 500 μL of the salivary sample was removed after administering the dose and diluted with 0.25% *w*/*v* SLS solution set to pH 6.8 at 4.5 mL [8]. 

### 3.9. Greenness of HPLC Method

The greenness was estimated using AGREE^®^ software v0.5 beta (Universida de Vigo, Vigo, Spain), which was available in the online repository [30]. Various parameters associated with the method such as the sample size, steps involved, sample transformation level, derivatization, sample analysis per hour, organic phase ratio, amount of toxic materials, use of energy and safety of operator were assessed. The scores as well as penalties are designed to qualify any technique with respect to the human working environment and geosafety [39]. The penalty points from these parameters were deducted according to the preset criteria of penalties and the results were presented as a pictogram. At the core of the spherical pictogram, the attained greenness score is displayed after subtracting the penalty points. The more the core color in the pictogram is green or close to it, the more it is closer to environmental safety and vice versa.

### 3.10. Statistical Analysis

The calculation of all the system suitability and method validation parameters were analyzed statistically using IBM SPSS v.20 software. The analyte concentrations in terms of percentage, mean and standard deviation values were also determined.

## 4. Conclusions

In the current study, we introduced a novel HPLC method for the simultaneous measurement of TBN and LGN from dosage form and saliva of volunteers, which previously was not reported. The system suitability was confirmed from the reasonable values of the tailing factor for the drugs, which were less than 2.0 with an absence of shouldering and elongated tail of the peaks, whilst the theoretical plate count was 5598 and 4339 for TBN and LGN, respectively. The resolution between the peaks was 2.98. The outcomes of validation in accordance with the ICH guidelines demonstrated that the method was linear, accurate, precise and robust at the concentrations prepared in the study. The LOD and LOQ values confirmed that both drugs were detectable as well as quantifiable. The overall RSD (%) values of all the experimental runs were less than 2%, which is generally considered good. The applied method to estimate the drugs simultaneously in dosage form and volunteers’ saliva did not depict any change in the peak, indicating the specificity of the method. The greenness of the developed method from the AGREE software was found to be 0.83. Conclusively, the method was linear, simple, sensitive, accurate, precise, robust and specific for the analytical determination of tibezonium iodide and lignocaine hydrochloride in dosage form and saliva.

## Figures and Tables

**Table 1:** System suitability, linearity, LOD and LOQ of the devised method for simultaneous detection of TBN and LGN.

Parameters	Tibezonium Iodide	Lignocaine Hydrochloride
System Suitability Parameters		
Retention time (min)	4.20 ± 0.28	2.33 ± 0.18
Tailing factor	1.86	1.32
Peak area	70.32 ± 0.74	98.97 ± 0.84
Theoretical plates (USP)	5598	4339
Resolution	2.591	---
Linearity Parameters		
Linear function	y = 26.321x + 0.7419	y = 1.9737x − 0.5297
Regression coefficient (r^2^)	0.9995	0.9992
Linearity range (µg/mL)	0.14–10.08	0.28–20.18
LOD (µg/mL)	0.011	0.115
LOQ (µg/mL)	0.037	0.384

**Table 2:** Percentage recovery indicating the accuracy of the devised method for simultaneous detection of TBN and LGN.

Percent Level	Tibezonium Iodide				Lignocaine Hydrochloride			
	Theoretical Content (µg/mL)	Amount Recovered (µg/mL)	Recovery (%)	RSD (%)	Theoretical Content (µg/mL)	Amount Recovered (µg/mL)	Recovery (%)	RSD (%)
80%	8.96	8.91	99.34	1.46	4.48	1.83	100.12	0.75
100%	11.2	11.15	100.01	1.72	5.6	2.56	99.05	0.65
120%	13.44	14.44	100.50	1.69	6.72	3.42	99.74	0.90

**Table 3:** Response of drugs indicating precision of the devised method.

Percent Level	Tibezonium Iodide				Lignocaine Hydrochloride			
	Percent Precision	Intraday RSD (%)	Percent Precision	Interday RSD (%)	Percent Precision	Intraday RSD (%)	Percent Precision	Interday RSD (%)
80%	99.34	1.83	98.63	1.75	100.42	0.96	99.89	1.46
100%	100.03	1.61	99.14	0.65	99.05	0.48	99.19	1.72
120%	100.10	1.65	99.01	0.90	99.84	0.77	98.84	1.69

**Table 4:** Response of TBN and LGN indicating the robustness of the developed HPLC method.

Parameters	Tibezonium Iodide Content % (RSD %)	Lignocaine HCl Content % (RSD %)
Optimal condition		
1.00 mL/min, 35 °C, pH 4.5	99.83 (1.52)	98.46 (0.65)
Flow rate variation		
0.85 mL/min	97.79 (1.24)	99.57 (0.91)
1.15 mL/min	98.11(1.86)	98.36 (0.62)
Temperature variation		
30 °C	98.60 (0.22)	99.93 (0.31)
40 °C	98.37 (0.29)	99.50 (0.24)
pH variation		
pH 4.0	99.19 (1.91)	100.42 (1.78)
pH 5.0	100.02 (1.81)	99.26 (1.91)

## Data Availability

The authors confirm that data are contained within the article.

## Sample Availability

Samples of the compounds will be available from the authors.
